# An Examination of Dr. Crawford's Theory of Heat and Combustion

**Published:** 1781-02

**Authors:** 


IV. *An Examination of Dr.* Crawford's *Theory of Heat and Combuſtion. By* William Morgan. 8vo. London. 72 *Pages.*

DR. Crawford, in this writer, meets with an able opponent. Mr. Morgan has examined his celebrated theory with freedom, and ſome of his objections appear to merit the Doctor's moſt ſerious attention.

He divides his performance into four ſections. In the firſt he gives a general account of Dr. Crawford's theory. With this we ſuppoſe our readers to be already acquainted; and therefore ſhall only obſerves, that Mr. Morgan has aſcribed to the Doctor diſcoveries, which, though they would do him honour, do not in reality belong to him. The theory of *abſolute and ſenſible heat* he has profeſſedly taken from Dr. Black and Dr. Irvine; that of "the power of phlogiſton to "diminiſh the quantity of fire or *abſolute heat* "in bodies," is all that he has pretended to.

In the ſecond ſection Mr. Morgan examines the experiments on which Dr. Crawford has founded his theory. Previous, however, to this he attacks Dr. Irvine's propoſition concerning the capacities of bodies for containing heat. He ſeems rather inclined to draw a concluſion the very reverſe of of Dr. Irvine's, viz. "that the capacity of a "body is greater in proportion as it is more "readily heated." His objection, however, is drawn from an analogy with saline solutions, which we do not apprehend to be just.

The arguments he has urged against the Doctor's experiments are of greater weight; Mr. Morgan has repeated many of them more than once, and finds the results to be different, not only from those of Dr. Crawford's, but from one another. Hence he infers the great difficulty of presenting these nice enquiries with sufficient accuracy; and shews how precarious any theory must be of which they are the foundation.

We acknowledge the justness of Mr. Morgan's remarks, though we cannot consider them as absolutely decisive on this point. Because a subject is difficult, it does not prove that it ought not to be enquired into; it can only be inferred, that greater attention and accuracy are necessary in the experimenter. Subjects as delicate as that in question have been prosecuted by philosophers, and the conclusions drawn from the experiments are nevertheless universally acknowledged. Absolute precision in these cases cannot be obtained, and we must content ourselves with a close approximation. The experiments should be be carefully repeated, not once or twice only, but a great number of times, and the conclusions taken from the mean of the several results.

In these cases too there are usually a variety of minute circumstances that are apt not to be sufficiently attended to, but which may yet make a very considerable difference in the events. Mr. Morgan, for example, has noticed the heat of the room at the beginning of his experiments, but does not seem to have paid any regard to the change it underwent while he was prosecuting them; yet this might probably have been one cause of the variations which he observed in the results of his trials. Similar objections seem also to lie against those of Dr. Crawford, as our author has justly observed.

Mr. Morgan objects with reason to the Doctor's method of ascertaining the heat of the mixture at the bottom of the vessel: "If (says he) "we examine these instances where the heated "metallic calces are mixed with cold water, we "shall instantly find the mixture hotter at the "bottom than at the top of the vessel. By "plunging the thermometer into the midst of "the calx *(at the bottom)* it does not denote the "heat which has been communicated to the "water, but that which the calx still retains."

He

He objects likewise, with reason, to his "taking "the arithmetical mean between the heat at the "surface and at the bottom of the vessel; that "not being always the truth." We do not, however, imagine that these objections are sufficient to invalidate Dr. Irvine's rule.

In the third section the author examines Dr. Crawford's propositions; he objects to the experiments on which they are founded, and affirms that on the most careful trials he found no difference in the absolute heats of the various kinds of air. This indeed is the most *tender* part of the Doctor's system, and we acknowledge the *extreme difficulty*, perhaps *impossibility*, of determining with any degree of accuracy, the different capacities of these very rare substances. We are told, however, that Dr. Crawford and his friends object to Mr. Morgan's thermometers, as not being sufficiently sensible. We learn also that the ingenious Abbé Fontana has repeated the experiments on phlogisticated and dephlogisticated air, and is satisfied of the truth of Dr. Crawford's conclusions. We are likewise informed that Dr. Crawford has repeated these experiments before several gentlemen who had doubted their accuracy, and that they became converts to his doctrine.

Mr.

Mr. Morgan next proceeds to examine the Doctor's propofition; "That the capacities of "bodies are diminifhed by the addition of "phlogifton, and increafed by its feparation:" and here we cannot but obferve, that he feems to have miftaken the author's meaning. The Doctor no where afferts that a phlogifticated body neceffarily contains lefs abfolute heat than one void of the former principle; he even gives an inftance to the contrary in the cafe of *blood* and *water*. His affertion is fimply as in the propofition above quoted. He has fupported that propofition by a number of experiments; and it adds fomething to its confirmation, that Mr. Elliot, with great fagacity, difcerned the fame thing *à priori* from the common analogy of Nature. It is further demonftrated by a variety of experiments made by the ingenious Mr. Kirwan, which may be feen in Mr. Magellan's excellent treatife on fire. Though therefore fpirit of wine, and fome other phlogifticated fubftances, contain more abfolute heat than water, we apprehend it does not affect the truth of the propofition in queftion. Other fubftances, befides phlogifton, may have a power of affecting the abfolute heat of bodies, either by leffening or enlarging it. It is fufficient in the prefent cafe if thefe fubftances have their capacities feverally altered, by varying the quantities of their phlogifton; the contrary of which by no means appears from Mr. Morgan's experiments.

In the laft fection, the author examines Dr. Crawford's application of his theory to phænomena. It follows from that theory, that air combined with phlogifton, ought to grow hotter than dephlogifticated air, when both are placed in the fame temperature. But Mr. Morgan could find no difference between them in this refpect; whether his thermometer was fufficiently accurate we cannot determine, but we remember an experiment of Mr.Cavallo's, which feems to make againft our author's conclufion: he found that the heat of a candle decreafed otherwife than in the duplicate ratio of the diftance: it was much greater near the flame than in that proportion. He was at a lofs to account for this phænomenon; but it would feem to have proceeded from the greater quantity of phlogifticated air immediately furrounding the flame; which, therefore, by Dr. Crawford's theory, was fufceptible of greater fenfible heat.

Several of our author's fubfequent remarks appear to be judicious and well founded; but as we have already extended this article to a great length, length, we muſt refer the reader, who is curious in theſe matters, to the performance itſelf. It muſt be acknowledged, that Dr. Crawford's theory is rendered doubtful by our author's ingenious ſtrictures; and that till theſe objections are removed, it can ſcarce be conſidered in any other light than that of an ingenious hypotheſis. As this controverſy involves a queſtion of great importance in philoſophy, we hope it will have the effect of ſtimulating the ingenious to clear up the truth by further experiments. The world will be indebted for that ſervice to Mr. Morgan; and we learn with pleaſure, that Dr. Crawford has already made ſome progreſs in this way.

