# Errata

**Published:** 1781-06

**Authors:** 


					-p. 109, J. 13,
. j c
for obferves read obierve.?p. no, 1. 12, J or prelenting read prolecutmg.
?p. Ill, !? 22, for ihftahtly read conftantly.?p. 113, 1. 27, for en-
larging read, incieafing.?p. 114, 1. 12, for thermometer was r*ad ther-
mometers were.?

				

